# Hevin/sparcl-1 as a potential biomarker and therapy in age-associated cognitive decline

**DOI:** 10.4103/NRR.NRR-D-25-00538

**Published:** 2025-09-03

**Authors:** Felipe Cabral-Miranda, Flávia C.A. Gomes

**Affiliations:** Instituto de Ciências Biomédicas, Universidade Federal do Rio de Janeiro, Rio de Janeiro, RJ, Brasil

Astrocytes, a major class of glial cells, have emerged as crucial regulators of synaptic function, neuronal homeostasis, and cognitive processes (Cabral-Miranda et al., 2024). These star-shaped cells not only provide structural and metabolic support to neurons but also actively participate in modulating synaptic transmission, neurovascular coupling, and inflammatory responses in the brain. Among the diverse array of astrocytic proteins implicated in neural function, Hevin (also known as SPARCL-1) has gained increasing attention due to its pivotal role in synapse formation and plasticity (Strunz et al., 2019; Gan and Südhof, 2020). Hevin has been shown to act as an extracellular matrix protein that promotes synaptic organization by bridging pre- and postsynaptic partners, thereby facilitating synaptic maturation and stability (Kucukdereli et al., 2011). Additionally, emerging evidence suggests that Hevin may influence neurodegenerative processes, particularly in Alzheimer’s disease (AD), where synaptic dysfunction is a hallmark feature. However, despite these advances, the precise mechanisms by which Hevin contributes to age-related cognitive decline and AD progression, including its interactions with amyloid-β, tau pathology, or neuroinflammatory pathways, remain poorly understood.

**Figure 1:**
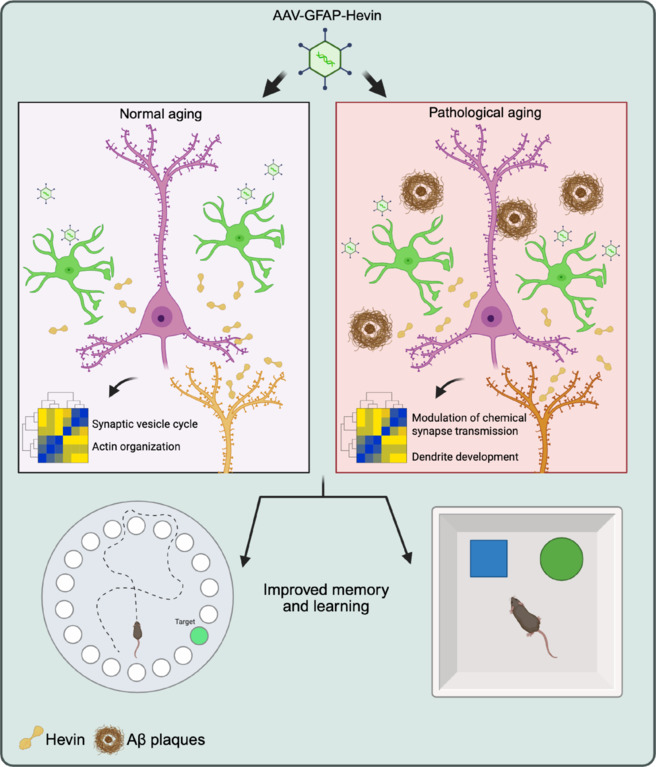
Mechanisms of Hevin action in normal and pathological aging. Effects of AAV-mediated delivery of Hevin in astrocytes (green) of aged wild-type (left panel) and APP/PSEN animals (right panel) modulating pre (pink) and postsynaptic (orange) neurons. In normal aging, Hevin modulates synaptic vesicle cycle and actin organization (heatmaps), contributing to healthy neuronal function. In pathological aging, astrocytic Hevin’s overexpression affects synapse transmission and dendrite development (heatmaps), which are associated with the progression of age-related neurodegenerative diseases, such as Alzheimer’s disease. Barnes Maze test (bottom left) and novel object recognition test (bottom right) are displayed to indicate improved memory and learning achieved in both models following astrocytic Hevin overexpression. In summary, by acting through distinct mechanisms/pathways, Hevin improves cognitive performance of aged wild-type and Alzheimer’s disease animal model. Created with with BioRender.com. AAV-GFAP-Hevin: Adeno-associated virus with glial fibrillary astrocytic protein promoter expressing Hevin; APP: amyloid precursor protein; Aβ: amyloid-beta; PSEN: presenilin.

To address this issue, we have recently investigated the impact of astrocytic Hevin/SPARCL-1 on cognitive decline in both normal aging and pathological conditions using the APP/PSEN mouse model of AD (Cabral‐Miranda et al., 2025). Our findings demonstrate that Hevin levels are significantly reduced in hippocampal astrocytes of both middle-aged wild-type and APP/PSEN mice, mirroring observations in human AD patients (Strunz et al., 2019). Using adeno-associated virus-mediated delivery in hippocampal astrocytes, Hevin overexpression was found to enhance synaptic integrity and improve cognitive performance in both aging and AD models without affecting amyloid-beta (Aβ) plaque deposition. These findings position Hevin as a promising therapeutic target for age-associated cognitive decline and neurodegenerative disorders, highlighting the importance of astrocytic support in maintaining synaptic function in those conditions. We evaluated human transcriptome datasets along with in-house immunohistochemistry analyses confirming reduced Hevin expression in AD patients and APP/PSEN mice astrocytes. Our adeno-associated virus approach directed increased levels of Hevin expression to hippocampal astrocytes in both wild-type and APP/PSEN mice, which abrogated cognitive decline in both models. Proteomic analysis revealed significant changes in synaptic protein expression, with increased colocalization of synaptophysin and Homer-1 in the hippocampus, suggesting improved synaptic connectivity. Conversely, Hevin overexpression did not affect Aβ plaque deposition, indicating its beneficial effects are mediated via synaptic mechanisms rather than Aβ clearance (**Figure 1**).

Hevin a secreted glycoprotein detectable in cerebrospinal fluid and peripheral blood, has emerged as a promising biomarker for AD and cognitive decline (Vafadar-Isfahani et al., 2012). Notably, post-mortem studies have revealed significantly decreased Hevin levels in the brains of AD patients, suggesting its potential role in disease pathogenesis. This finding raises the possibility that circulating Hevin levels in serum might reflect central nervous system changes, providing a clinically accessible biomarker. The strong correlation observed between cerebrospinal fluid and serum Hevin levels in preliminary studies supports this hypothesis, though further research is needed to establish whether serum concentrations reliably mirror brain tissue depletion (Vafadar-Isfahani et al., 2012). The diagnostic potential of Hevin is underscored by proteomic studies where it was identified as the top predictor in an AD biomarker panel. In initial validation, this panel demonstrated 85% sensitivity and 97% specificity, with subsequent confirmation in an independent cohort showing 93.3% sensitivity and 75.7% specificity (Vafadar-Isfahani et al., 2012). These robust performance metrics suggest Hevin could significantly improve current diagnostic protocols, particularly in distinguishing AD from other dementias and identifying at-risk individuals in pre-symptomatic stages. Future studies should investigate the temporal relationship between declining brain Hevin, cerebrospinal fluid/serum levels, and cognitive impairment progression. If validated, serum Hevin monitoring could enable earlier, less invasive AD detection while providing prognostic information to guide therapeutic decisions. Additionally, understanding the mechanisms behind Hevin depletion in AD may reveal novel therapeutic targets (Vafadar-Isfahani et al., 2012).

Regarding its therapeutic potential, further research should explore Hevin-based strategies such as gene therapy, recombinant protein administration, or pharmacological modulation to enhance astrocytic support and synaptic resilience. Unlike current AD therapies that focus narrowly on Aβ clearance, pleiotropic roles of Hevin in synapse formation, maintenance, and plasticity could address broader aspects of neurodegeneration. Importantly, these approaches may also benefit non-pathological brain aging. Age-related synaptic decline, characterized by weakened connectivity and reduced plasticity, shares molecular hallmarks with early AD (e.g., synaptic protein loss and glial dysfunction) but often progresses without frank pathology. By bolstering synaptic stability and astrocyte-neuron communication, Hevin modulation could mitigate age-related cognitive decline even in the absence of overt disease. This dual potential, for both delaying neurodegenerative progression and preserving function in healthy aging, positions Hevin as a unique candidate for next-generation interventions targeting synaptic vulnerability across the aging continuum (**Figure 1**). Moreover, evaluating how Hevin compares with other astrocyte-derived factors, such as thrombospondins and glypicans (Verkhratsky et al., 2023) in neurodegenerative contexts could provide complementary or even better therapeutic targets. Recent evidence indicates that the composition of the milieu secreted by astrocytes may influence the physiopathology of AD (Verkhratsky et al., 2023), which is in line with our findings extending also to age-associated cognitive decline, as observed following Hevin overexpression in middle-aged wild type animals. Interestingly, most studies implicated astrocytes with reduced Aβ levels, which correlated with better cognitive outcomes and less neuroinflammation (Verkhratsky et al., 2023). Our findings support a new concept in this field, as we observed that the improvements in cognition following astrocytic reprogramming in middle-aged APP/PSEN animals did not correlate with altered Aβ burden. In those lines, recent studies are investigating AD treatments beyond Aβ targeting and although Aβ immunotherapies like aducanumab and donanemab show some promise, their impact on cognitive decline remains modest and alternative approaches, such as neuroinflammation mediators and vascular health are gaining more attention (Mullane and Williams, 2020), placing astrocytes as potential novel players in the development of novel strategies for dementia

While our study provided important insights into the role of Hevin in synaptic regulation, several key questions remain unresolved. Specifically, we could not fully elucidate how Hevin interacts with synaptic organizers such as neurexins, neuroligins, or other adhesion molecules, nor could we pinpoint the exact signaling pathways impacted by its overexpression in the context of aging and AD. Previous work has established that Hevin promotes glutamatergic synapse assembly by bridging neuroligin-1 and neurexin-1β, thereby stabilizing synaptic connections (Fan et al., 2020; Kang et al., 2022). However, conflicting evidence suggests Hevin may also operate independently of these canonical pathways, as one study demonstrated its ability to enhance excitatory synapse formation even in the absence of neurexin/neuroligin interactions (Gan and Südhof, 2020). This duality underscores the multifaceted role of Hevin in synaptic plasticity and highlights the complexity of its mechanistic contributions. Our proteomic analysis revealed that Hevin overexpression in the hippocampus of APP/PSEN1 mice significantly altered a cluster of proteins involved in chemical synaptic transmission (Cabral-Miranda et al., 2025; **Figure 1**). Strikingly, these same proteins exhibited strong positive correlations with Hevin levels in post-mortem AD patient brains, suggesting that synaptic regulatory function of Hevin is clinically relevant to AD pathophysiology. In contrast, when examining middle-aged wild-type mice, Hevin overexpression impacted distinct protein clusters associated with synaptic vesicle formation and transport and cytoskeleton organization, changes that paralleled improved cognitive performance in behavioral assays such as the Barnes maze and novel object location tests (Cabral-Miranda et al., 2025; **Figure 1**). These divergent effects in pathological (APP/PSEN1) versus non-pathological aging models imply that synaptic targets of Hevin may vary depending on the disease state of the brain, potentially acting as a compensatory mechanism in AD while optimizing vesicular dynamics in healthy aging.

The translational implications of these findings are significant. If synapse-modulating functions of Hevin can be harnessed therapeutically, it may offer a dual benefit: preserving cognitive function in normal aging and counteracting synaptic loss in neurodegenerative conditions. Future studies should prioritize mapping the interactome of Hevin under varying physiological and pathological conditions, as well as investigating whether its effects are region-specific (e.g., hippocampal versus cortical synapses). While our findings show that Hevin acts independently of amyloid-β clearance, further investigation into its potential interactions with other AD-related pathways, such as amyloid-β metabolism, tau propagation, or neuroinflammation, could reveal novel combination therapies.

Ultimately, a deeper understanding of molecular mechanisms of Hevin and astrocyte role may pave the way for targeted interventions to mitigate synaptic dysfunction across the aging-neurodegeneration continuum.
